# Progress in Research of Flexible MEMS Microelectrodes for Neural Interface

**DOI:** 10.3390/mi8090281

**Published:** 2017-09-18

**Authors:** Long-Jun Tang, Ming-Hao Wang, Hong-Chang Tian, Xiao-Yang Kang, Wen Hong, Jing-Quan Liu

**Affiliations:** 1 National Key Laboratory of Science and Technology on Micro/Nano Fabrication Laboratory, Shanghai Jiao Tong University, Shanghai 200240, China; tanglongjun@sjtu.edu.cn (L.-J.T.); m.h.wang89@sjtu.edu.cn (M.H.W.); tianhch@xdps.com.cn (H.-C.T.); xiaoyang.kang@epfl.ch (X.-Y.K.); nikkihong@sjtu.edu.cn (W.H.); 2 Key Laboratory for Thin Film and Micro fabrication of Ministry of Education, Shanghai Jiao Tong University, Shanghai 200240, China; 3 Collaborative Innovation Center of IFSA, Department of Micro/Nano-Electronics, Shanghai Jiao Tong University, Shanghai 200240, China

**Keywords:** MEMS, microelectrodes, neural interface, conducting polymer, nanotechnology

## Abstract

With the rapid development of Micro-electro-mechanical Systems (MEMS) fabrication technologies, many microelectrodes with various structures and functions have been designed and fabricated for applications in biomedical research, diagnosis and treatment through electrical stimulation and electrophysiological signal recording. The flexible MEMS microelectrodes exhibit excellent characteristics in many aspects beyond stiff microelectrodes based on silicon or metal, including: lighter weight, smaller volume, better conforming to neural tissue and lower fabrication cost. In this paper, we reviewed the key technologies in flexible MEMS microelectrodes for neural interface in recent years, including: design and fabrication technology, flexible MEMS microelectrodes with fluidic channels and electrode–tissue interface modification technology for performance improvement. Furthermore, the future directions of flexible MEMS microelectrodes for neural interface were described, including transparent and stretchable microelectrodes integrated with multi-functional aspects and next-generation electrode–tissue interface modifications, which facilitated electrode efficacy and safety during implantation. Finally, we predict that the relationships between micro fabrication techniques, and biomedical engineering and nanotechnology represented by flexible MEMS microelectrodes for neural interface, will open a new gate to better understanding the neural system and brain diseases.

## 1. Background

**Figure 1:**
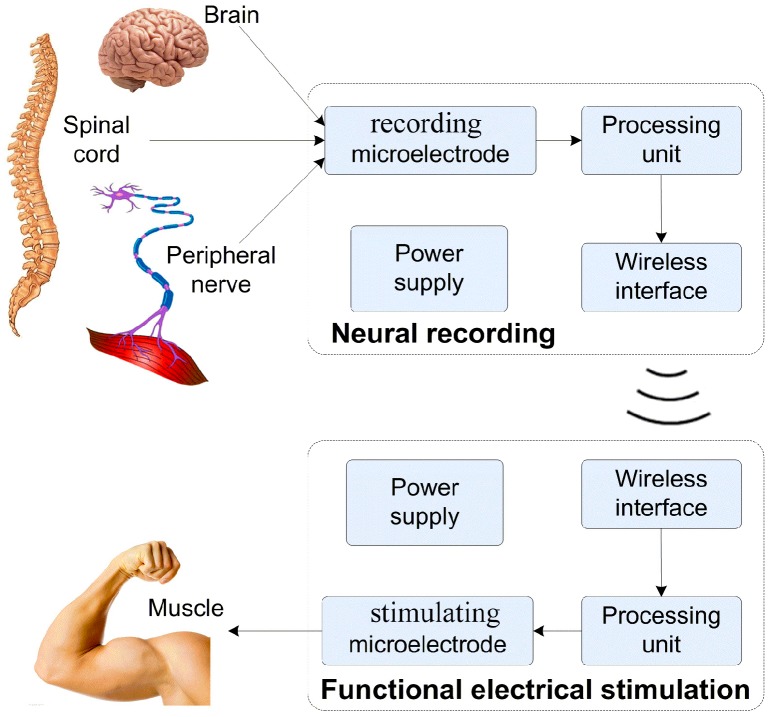
Structural illustration of implantable artificial nerve system for paralysis rehabilitation.

The function of microelectrodes in neural interfaces can be categorized as neural signal recording and neural stimulating. Figure 1 exhibits a representative configuration of an implantable artificial nerve system for paralysis rehabilitation that may bring hope to patients who have suffered a spinal cord injury. It can be seen that, in the whole system, the microelectrodes (recording microelectrodes or stimulating microelectrodes) play a key role in interconnecting biological tissues (brain, nerve, or muscle) with implantable devices. For example, command signals generating from the cerebral cortex are acquired through the recording (or measuring) microelectrodes, which are analyzed and processed by the processing unit. The obtained information is further processed to generate stimulating signals, and then transmitted through the stimulating microelectrodes to the nerve or muscle, for a diverse type of stimulations. For chronic implantation, the microelectrodes act as the tissue–machine interface that make direct contact with biological tissues, and may cause immunological reaction, mechanical damage, and neuronal degradation to tissues, on the one hand. On the other hand, the properties of the microelectrodes can largely effect the quality of the record and stimulation of neural signals, such as signal-to-noise-ratio, stability, resolution, and accuracy. All these issues of neural interfaces mentioned above need to be addressed for practical applications.

**Figure 2:**
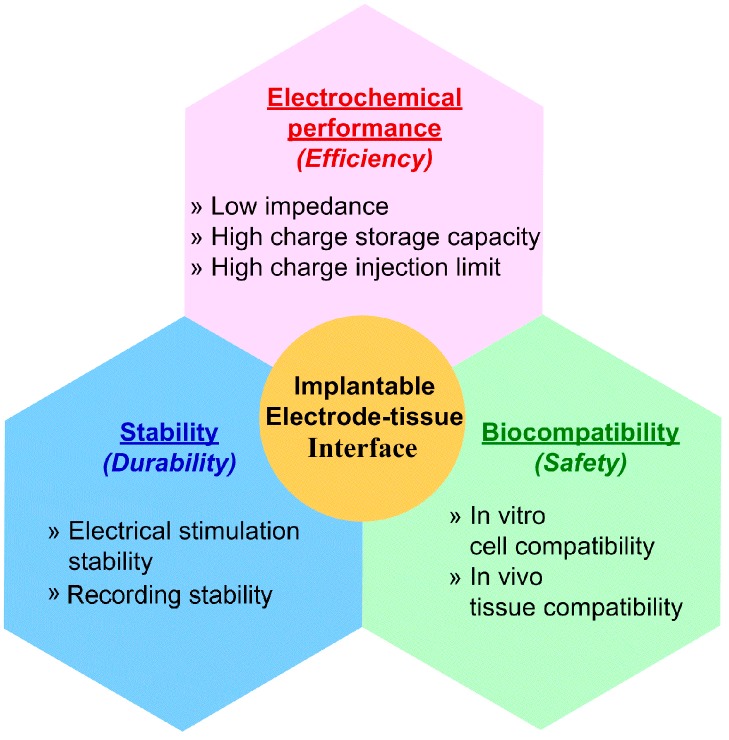
Characteristics of ideal implantable electrode–tissue interface.

With the rapid development of micro fabrication technology, biomedical devices can be manufactured in a considerably tiny and structurally diverse manner, which minimizes the damage during and after implantation in both the short and the long term [1]. Nowadays, some dense electrode arrays and tenuous electrodes are developed to undertake complex and precise electrophysiological research by providing excellent spatial selectivity and low power consumption. The problem is that a smaller size, that certainly lowers the damage to the tissue, would inevitably damage the performance and safety of the electrodes [2], because the decrease in the size of the electrode leads to an increase in impedance and a drop in charge storage capacity (CSC), which, as a result, means poor recording signal quality and a high stimulating current that may damage tissue. Considering this fact, the interface material plays a significant role in improving the electrode performance. Many efforts have been made to develop more ideal electrode–tissue interface materials with properties including: electrical property containing low impedance, high CSC and high charge injection limit stability for long term work or implantation without significant property variation; biocompatibility ensuring direct contact with tissue without inducing severe tissue response, toxicity or even necrosis [3,4,5]. The characteristics of an ideal implantable electrode–tissue interface are shown in Figure 2.

### 1.1. Flexible Micro-Electro-Mechanical Systems (MEMS) Microelectrodes for Neural Interface

One of the most significant components of the artificial prostheses is the microelectrodes which act as the tissue–machine interface [6,7,8]. To functional well in live muscle and nerve tissue, the biomedical microelectrodes should meet such requirements as: (1) miniature dimension that minimizes the tissue damage and power consumption; (2) excellent performance that ensures effective operation of the prostheses device and; (3) good biocompatibility that guarantees relatively long term implantation without inducing severe immune response. Complying with these disciplines for biomedical electrodes as mentioned above, various kinds of microelectrodes were developed to execute electrical stimulation and electrophysiological signal recording. Among these manifold electrodes, Michigan neural probes and Utah electrodes array have been widely used in central nerve prostheses applications [9,10], while LIFE electrodes are usually applied in peripheral nerve and intramuscular researches [7,11]. However, a key technical challenge of these rigid indwelling electrodes is the mechanical mismatch between the compliant brain tissue and the electrode substrate [12,13,14], which can cause tissue damage and reduce the ability for long term recording. The typical elastic modulus for a rat cerebral cortex is from 0.03 to 1.75 kPa [15], while for silicon is approximately 200 GPa. These problems may cause inflammation, tissue reactivity, scar formation and accessory mechanical damage [16,17,18]. While the use of ultrathin film “flexible” silicon probes can partially alleviate the mechanical mismatch that exists at the tissue–electrode interface, deformation induced from localized stresses readily leads to material rupture [19]. Due to the inherent brittleness and tendency of silicon to fracture, as well as its limited flaw tolerance, overall electrode integrity can be compromised during tissue loading. The mismatch of stiffness between implantable devices and tissues can also be addressed by employing thin film with lower elastic moduli (200 GPa for silicon), for example, polyimide (2.3–8.5 GPa), parylene (~3 GPa), polydimethylsiloxane (PDMS) (0.36–0.87 MPa), and liquid crystal polymer (LCP, ~10.6 GPa). When constructing flexible, insertable microelectrodes, it is desirable to use electrode substrates with lower elastic moduli than silicon which can decrease the micromotion and alleviated tissue encapsulation of the implant. In this review we define electrode substrates with elastic modulus smaller than 10 GPa and thickness smaller than 200 μm as flexible.

It is a significant symptom that should be confronted that only electrical interaction between electrodes and muscle or nerve tissue without nutrition factor delivery would eventually lead to denervation-induced skeletal muscle atrophy [20,21,22]. Considering this fact, microelectrodes integrated with micro channels for fluidic drug delivery were developed in recent years [23,24,25]. As the majority of these studies focused on flexible electrodes made of parylene, polyimide (PI) and polydimethylsiloxane (PDMS) [26,27,28], the flexible microelectrodes integrated with micro channels were intensively reviewed [29,30].

### 1.2. Electrode–Tissue Interface of Flexible MEMS Microelectrodes for Neural Interface

In order to upgrade electrode performance of implantable Micro-Electro-Mechanical Systems (MEMS) microelectrodes, the ideal implantable electrode–tissue interface materials should satisfy requirements, including excellent electrochemical performance, good stability and biocompatibility. Therefore, many electrode–tissue interface materials have been developed to meet the practical demands. Nowadays, the most widely used electrode–tissue interface material remains noble metals, such as platinum, gold, iridium, tungsten and their alloys. These metals are chosen to be electrode–tissue interface because of their excellent chemical stability, which implies that they are able to be implanted in tissue without serious erosion. However, these materials are not suitable for microelectrodes with small-area sites due to their low charge injection limits (0.05–0.3 mC/cm^2^) [31], and few options for dramatic impedance improvement [32]. Some electrode–tissue interfaces were processed with porous structure and special profile to form a rough surface, which would consequently improve the effective surface area of electrode sites. In recent years, newly rising carbon nano materials, including carbon nanotubes and graphene, act as the electrode–tissue interface to improve MEMS microelectrode performance for their excellent multi-functional properties. Although the carbon nano materials have an extremely large specific area and excellent electrochemical performance that others do not match, the drawbacks of a poor bonding effect with electrode substrate and the probably induced nanotoxicity by litters in tissue, largely limit their applications in the electrode–tissue interface.

**Figure 3:**
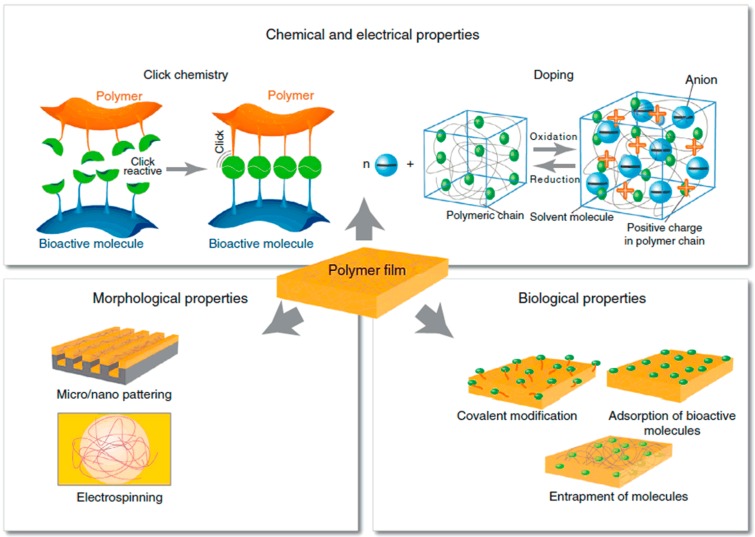
Schematic illustration of characteristics of conducting polymers, reprinted from [33] with permission of Elsevier, Copyright 2013.

Conducting polymers have also attracted much attention and have been broadly applied in many aspects of research in the biomedical domain for their unique characteristics, which include: low electrochemical impedance, high CSC, favorable plasticity (the performance can be optimized by doping counting ions), electrostriction (the volume can be changed by applying voltage) and biocompatibility. Simultaneously, conducting polymers are capable of meeting the requirements of electrode–tissue interface material. In addition, as shown in Figure 3, conducting polymers possess characteristics including: modification through doping, electrically controlled drug release, molding through micro-nano processing and electro-spinning and surface modification by biochemical molecules. As two kinds of conducting polymers that are widely used as electrode–tissue interface, poly (3,4-ethylenedioxythiophene) (PEDOT) exhibits better performance than polypyrrole (PPy) in electrical stimulation (better electrochemical performance) and cell culture (longer neurites growth) [33].

## 2. Research Progress

### 2.1. Flexible MEMS Microelectrodes for Neural Interface

With the rapid development of MEMS fabrication technologies, researchers have been developed with various kinds of biomedical microelectrodes applied on electrical stimulation and electrophysiological signal recording for paralysis recovery. Among these many microelectrodes, Michigan neural probes and Utah electrode array are widely applied in central nerve system research as stiff MEMS microelectrodes for neural prosthesis. Many research efforts have been devoted to developing novel flexible MEMS microelectrodes for their excellent multi-functional characteristics, as compared to stiff microelectrodes, including their lighter weight, smaller volume, more effective conforming to neural tissue and lower fabrication cost. For example, Mercanzini et al. of École polytechnique fédérale de Lausanne (EPFL) described the fabrication of a novel, flexible, polyimide neural probe with two layers of platinum electrodes providing mechanical flexibility, high quality electrical characteristics and excellent biocompatibility. Two layers of platinum electrodes were used which greatly reduced the size of the neural probes, thereby limiting the insertion damage [34]. Xu et al. of University of Southern California designed and developed a flexible parylene-C based neural probe which can be easily micro-machined to the desired dimensions. The implantation procedure of the flexible parylene probe was developed and optimized both in brain tissue phantom and in vivo with a sham device. Immunohistochemistry (IHC) staining post-implantation of the sham probe was used to evaluate immune responses to the probe. The result of the GFAP stain indicated that no extraordinary activation of glia cells was observed around the implanted site and surrounding neurons did not retract from implantation sites as indicated by the NeuN stain [35]. Owning to these advantages, the flexible MEMS microelectrodes for neural interface have generated extensive attention and have been considered to have broad prospects for development in the future. The flexible MEMS microelectrodes mainly include: wire electrode, thin film electrode and mesh electrode.

**Figure 4:**
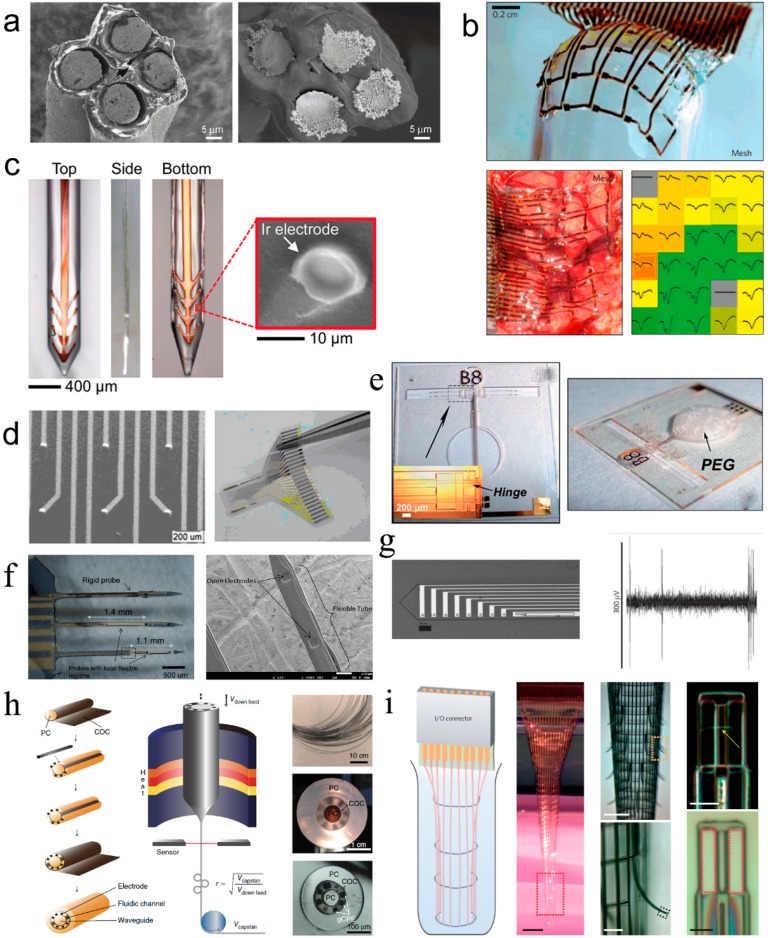
Research progress of flexible MEMS microelectrodes for neural interface, (**a**) Tetrode composed of four micro wire electrodes, reprinted from [36] with permission of Elsevier, Copyright 2009; (**b**) Thin film microelectrode array with silk fibroin covered as substrate reprinted from [37] with permission of Nature Publishing Group, Copyright 2012; (**c**) Brain-penetrating flexible electrodes with biodegradable silk as substrate, reprinted from [38] with permission of John Wiley and Sons, Copyright 2013; (**d**) Thin film microelectrode with 3D raised hemispherical electrode sites, reprinted from [39] with permission of Springer, Copyright 2011; (**e**) Three-dimensional flexible microprobe array with electrostatic actuation, reprinted from [40] with permission of Royal Society of Chemistry , Copyright 2011; (**f**) Hybrid silicon–parylene neural probe with locally flexible regions, reprinted from [41] with permission of Elsevier, Copyright 2014; (**g**) Flexible cortical probes with shape memory polymer as substrates, reprinted from [42] with permission of John Wiley and Sons, Copyright 2012; (**h**) Multifunctional flexible fiber microelectrode integrated with optical waveguide and microfluidic channels, reprinted from [43] with permission of Nature Publishing Group, Copyright 2017; (**i**) Three-dimensional macroporous nanoelectronic brain probe, reprinted from [44] with permission of Nature Publishing Group, Copyright 2015.

Ferguson et al. of University of Minnesota used tetrode (four channels microwire electrodes) to record neural signals on freely moving animals in 2009 [36]. As shown in Figure 4a, the tetrode was composed of four Ni-Cr alloy microwires with a diameter of 12.7 μm and insulated with polyimide on the surface. The microwire electrodes were cut to expose the electrode sites which were further electrodeposited with gold to improve their electrochemical performance. The advantages of the microwire electrodes were that they could be conveniently fabricated and were suitable for cortical implantation with little tissue damage. However, the microwire electrodes were assembled by hand and not suitable for large scale production.

Kim et al. of University of Illinois at Urbana-Champaign developed a thin film microelectrode array based on polyimide for electrocorticogram (ECoG) recording in 2010. The microelectrode array was reinforced by silk fibroin, which acts as a biodegradable substrate, to improve the conformal attachment on the brain tissue surface [37]. As displayed in Figure 4b, the thickness of the 5×6 grid-like thin film microelectrode array was approximately 2.5 μm, and the dimension of the electrode site was 500 μm × 500 μm. Moreover, it can be observed from Figure 4b that the thin film microelectrode array tightly attached on the sphere surface when the silk fibroin dissolved. The biodegradable silk fibroin surface coating facilitated the conformal cover of the mesh electrode on the rough surface of brain. Additionally, the mesh electrode could be fabricated thinner and the electrode sites could be designed smaller to further improve the conformal attachment on brain and accuracy of neural recording.

Tien et al. of Tufts University proposed silk fibroin as a multifunctional material for fabricating brain-penetrating flexible electrodes that combine dynamic mechanical properties with the capacity for local release of sensitive therapeutics [38]. Silk is both biocompatible and biodegradable, with tunable, hydration dependent mechanical properties. Through controlled crystallization of the protein, it can be programmed to dissolve immediately upon exposure to liquid, or to persist for years in vivo. As shown in Figure 4c, a layer-by-layer casting technique was used to coat polyimide based, thin film neural probes with silk fibroin. Uncoated, 3 layer, and 6 layer silk-coated probes were inserted into an agar hydrogel mechanical brain phantom in order to test the efficacy of the silk coating for facilitating the flexible electrode insertion into the cortex. The results showed 6 layer silk-coated probe penetrated the gel and remained straight during the entire insertion.

Rui et al. of Shanghai Jiao Tong University developed a flexible 3D microelectrode array with raised hemispherical electrode sites in 2011 [39]. The electrode sites were arranged in 5×5 array, which had a diameter of 50 μm, and the gap between two adjacent electrodes was 600 μm. As exhibited in Figure 4d, compared with flat electrode sites, the micro scale 3D hemispherical electrode sites facilitated the contact to nerve tissue, increased the effective contact area and reduced the interfacial resistance. Thus, the electrical stimulation and neural signal recording performance was improved.

Chen et al. of National Tsing Hua University designed a novel three-dimensional flexible microprobe to record neural signals of a lateral giant nerve fiber of the escape circuit of an American crayfish [40]. As shown in Figure 4e, an electrostatic actuation was adopted to fold the planar probes into three-dimensional neural probes with arbitrary orientations for neuroscientific applications. The novel fabrication technique based on SU-8 and parylene can decrease the micromotion and alleviated tissue encapsulation of the implant. With this flexible probe, they recorded neural signals of a lateral giant cell with a ratio of signal to noise as great as 30.22 ± 3.58 dB.

Kim and Xu of Wayne State University reported the development of flexible neural probes based on hybrid silicon–parylene structures to overcome the difficulty of implantation of soft probes and decrease the mechanical mismatch by the “local softness” concept [41]. Figure 4f showed a schematic of their hybrid silicon–parylene probe with a local flexible region. The majority of the probe, including the sharp tip, is made of rigid silicon except the area surrounding the electrodes, which was replaced by a flexible parylene tube. The implantation experiment of the probe into agarose gel demonstrated the whole probe can still be mechanically rigid, allowing easy implantation into a neural tissue. Ware et al. of University of Texas described a novel processing method using photolithography to pattern thin-film flexible electronics on shape memory polymer substrates with mechanical properties tailored to improve biocompatibility and enhance adhesion between the polymer substrate and metal layers [42]. As shown in Figure 4g, this work utilizes the tailorable significant modulus change of acrylic shape memory polymers that can be activated by physiological conditions. The produced cortical probes soften post-insertion to more closely match the modulus of cortical tissue. Devices are fabricated using a transfer-by-polymerization process that allows for full-photolithographically defined electrodes on temperature and solvent sensitive substrates. 

Park et al. of Massachusetts Institute of Technology developed a device composed of an optical waveguide, six electrodes and two microfluidic channels produced via fiber drawing as shown in Figure 4h. [43] The probes facilitated injections of viral vectors carrying opsin genes while providing collocated neural recording and optical stimulation. The miniature (<200 μm) footprint and modest weight (<0.5 g) of these probes allowed for multiple implantations into the mouse brain, which enabled opto-electrophysiological investigation of projections from the basolateral amygdala to the medial prefrontal cortex and ventral hippocampus during behavioral experiments. Fabricated solely from polymers and polymer composites, these flexible probes minimized tissue response to achieve chronic multimodal interrogation of brain circuits with high fidelity.

Xie and Lieber of Harvard University developed a three-dimensional macroporous nanoelectronic brain probe that combined ultra-flexibility and subcellular feature sizes to overcome the dimensional and mechanical mismatch of these probes with the brain tissue which limited their stability in chronic implants and decreased the neuron–device contact [44]. As shown in Figure 4i, the fabrication exploits conventional planar 2D lithography with a sacrificial layer that is etched to yield the free-standing macroporous nanoelectronic probe. The overall design of the mesh probe consists of longitudinal metal interconnects that are sandwiched between SU-8 polymer layers for passivation and transverse SU-8 polymer structural elements. In addition, transverse compressive strain elements are incorporated to generate positive transverse curvature and yield a cylindrical global probe structure, and local tensile strain elements in the supporting arms of each sensor device are incorporated to produce negative curvature, bending the devices away from the surface of the cylinder. The local geometry of the macroporous devices are designed to optimize the neuron/probe interface and to promote integration with the brain tissue while introducing minimal mechanical perturbation. The ultra-flexible probes were implanted frozen into rodent brains and used to record multiplexed local field potentials and single-unit action potentials from the somatosensory cortex. Significantly, histology analysis revealed filling-in of neural tissue through the macroporous network and attractive neuron–probe interactions, consistent with long-term biocompatibility of the device.

### 2.2. Flexible MEMS Microelectrodes with Micro Channels for Neural Interface

While the researchers focus on developing MEMS microelectrodes with smaller dimensions, more complex structures and denser electrode sites distribution, they are also devoting much effort to multi-functionalizing the MEMS microelectrodes. The nerve conduction and muscle contraction actions of denervated paralyzed nerve and muscle tissue could be restored by electrical/optical stimulation based on MEMS microelectrodes for the artificial neural system. However, long-term lack of supply of neurotrophic factors would eventually lead to denervation atrophy of nerve and muscle tissue, which would mean irreversible loss of natural conduction of the nerve system and the contraction function of muscle. Therefore, various types of MEMS microelectrodes integrated with micro channels for drug or light delivery have been developed. Based on the inherent electrical/optical stimulation and electrophysiological signal recording properties, the novel MEMS microelectrodes integrate functions of delivering fluidic drugs, nutritional factors and light to target nerve and muscle tissue sites.

**Figure 5:**
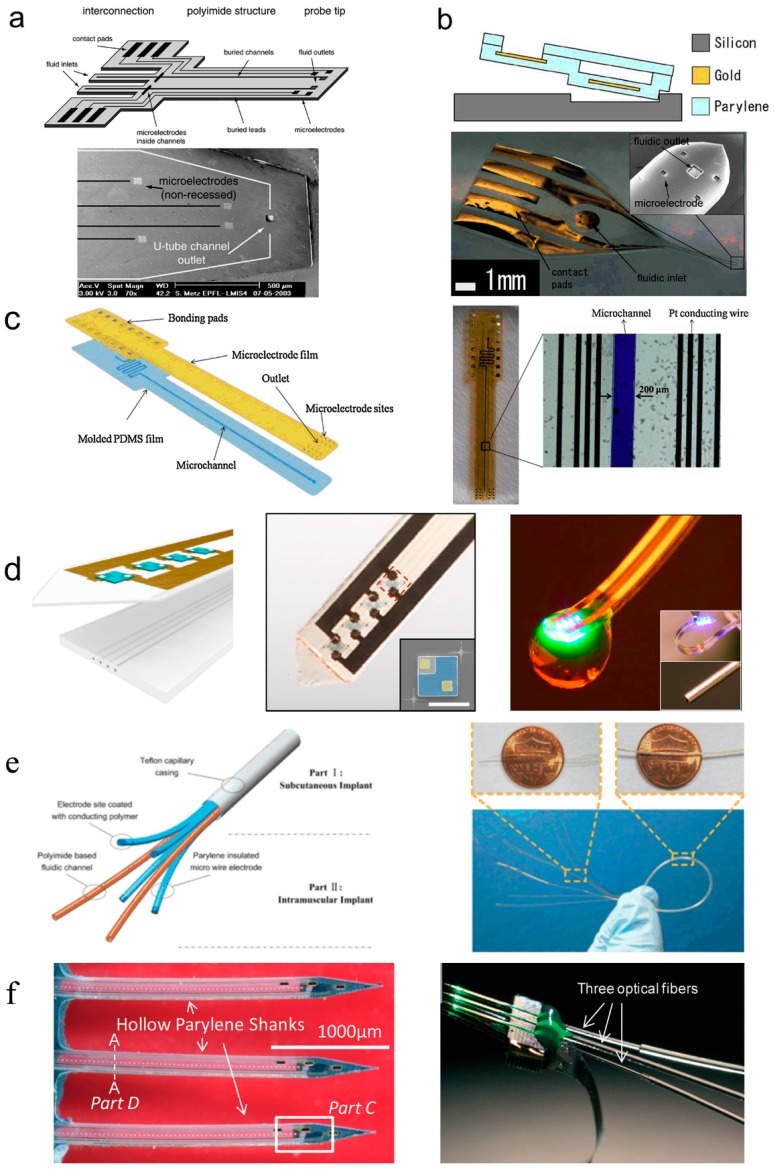
Research progress of flexible MEMS microelectrodes with fluidic channels for neural interface. (**a**) Thin film microelectrode array based on polyimide (PI), reprinted from [26] with permission of Elsevier, Copyright 2003; (**b**) Thin film microelectrode array based on Parylene, reprinted from [45] with permission of IEEE, Copyright 2006; (**c**) Thin film microelectrode array based on poly-dimethylsiloxane (PDMS), reprinted from [28] with permission of Elsevier, Copyright 2013; (**d**) Ultrathin, soft optofluidic probe based on PDMS, reprinted from [46] with permission of Elsevier, Copyright 2015; (**e**) Microelectrodes integrated polyimide micro fluidic channels and parylene micro wire electrodes, reprinted from [48] with permission of Elsevier, Copyright 2013; (**f**) 3D silicon neural probe with integrated parylene micro channels and optical fibers, reprinted from [49] with permission of Royal Society of Chemistry, Copyright 2015.

Metz et al. of EPFL developed flexible MEMS microelectrodes with fluidic channels based on polyimide (PI) in 2004 [26]. As displayed in Figure 5a, the thickness of the microelectrode array was 10~60 μm, and the dimension of electrode sites was 50 μm × 50 μm. The cross-section dimension of inner fluidic channels was 5 μm × 50 μm or 20 μm × 200 μm, and the cross-section dimension of fluidic channel exits was 30 μm × 30 μm or 50 μm × 50 μm. The thickness of the PI microelectrode array was relatively small, which was suitable for cortical implantation and neural recording. However, the one-sided distribution of electrode sites affected the functional scope of neural recording and stimulation.

Ziegler et al. developed flexible MEMS microelectrodes with fluidic channels based on parylene in 2006 [45]. As shown in Figure 5b, the thickness of the microelectrode array was 18 μm, and the dimension of electrode sites was 40 μm × 40 μm. The cross-section dimension of inner fluidic channels was 15 μm × 80 μm, and the cross-section dimension of fluidic channel exits was 100 μm × 100 μm. The parylene microelectrode array was quite thin, which benefited the conformal cover on brain cortex. However, the parylene thin film electrode was too thin to withstand the internal pressure produced by tissue motion, which might lead to the closure of the fluidic channel and disability of drug delivery.

Gao et al. of the Shanghai Institute of Micro-System and Information Technology (Chinese Academy of Sciences) developed flexible MEMS microelectrodes with fluidic channels based on poly-dimethylsiloxane (PDMS) in 2013 [28]. As shown in Figure 5c, the thickness of the microelectrode array was 125 μm. The cross-section dimension of inner fluidic channels was 50 μm × 200 μm. The thickness of the PDMS microelectrode array was relatively large, which was unfavorable for flexibility and conformal attachment on tissue.

Jeong et al. of University of Colorado developed ultrathin and soft neural probes based on PDMS for programmed drug delivery and photostimulation [46,47]. As illustrated in Figure 5d, the multichannel, soft microfluidic system in which two thin, narrow pieces of the elastomer PDMS bond together to form a set of four channels each with 10 µm × 10 µm cross-sectional areas in a platform that has a total thickness of 50 µm. These microfluidic probes are transparent and mechanically soft (modulus ~1 MPa; bending stiffness 13–18 N/m), thereby enabling both optical access and minimally invasive use in soft neural tissue. The former characteristic facilitates integration of microscale inorganic light-emitting diodes (µ-ILEDs) on a filament of polyethylene terephthalate (PET) with a thickness of 6 µm. These µ-ILEDs (lateral dimensions of 100 µm × 100 µm and 6.54 µm thick) provide spatially and temporally precise delivery of light adjacent to the outlets of the microfluidic channels. Active infusion of multiple drugs through these four individual channels can be controlled independently from the m-ILEDs. This system allows for tandem pharmacological and optogenetic manipulation of neural circuitry, with potential for application in optopharmacology where the use of light to activate compounds requires high spatiotemporal control of both drug and light delivery.

Tian et al. of Shanghai Jiao Tong University developed flexible MEMS microelectrodes integrated polyimide micro fluidic channels and parylene micro wire electrodes in 2014 [48,49]. As shown in Figure 5e, the integrated flexible microelectrode was composed of three parts: (1) the micro wire electrodes as electrical interfaces; (2) the PI capillaries (outer diameter of 110 μm and wall thickness of 10 μm) for fluidic drug release as chemical interfaces; and (3) the Teflon capillary (outer diameter of 650 μm and wall thickness of 140 μm) casing for packaging. The integrated microelectrode with drug delivery function was easy to fabricate and change parameters. More electrode sites and fluidic channels and smaller dimension of the electrode sites and fluidic channels were required to satisfy more complex and precise neural recordings and stimulations.

Kim et al. of Wayne State University report the development of a novel 3D neural probe coupled simply and robustly to optical fibers using a hollow parylene tube structure [50]. As illustrated in Figure 5f, the device shanks are hollow tubes with rigid silicon tips, allowing the insertion and encasement of optical fibers within the shanks. The position of the fiber tip can be precisely controlled relative to the electrodes on the shank by inherent design features. Preliminary in vivo rat studies indicate that these devices are capable of optogenetic modulation simultaneously with 3D neural signal recording.

Canales et al. of Massachusetts Institute of Technology present a class of neural probes fabricated from polymer, metal and composite materials by means of a thermal drawing process (TDP) traditionally employed in optical fiber production [51]. They successfully used the fiber probes for simultaneous optogenetic stimulation, neural recording and drug delivery in freely moving mice. Moreover, histological assessment of the tissue response showed that the fiber probes yielded stable multifunctional interfaces with the brain.

Various types of microfluidic neural probes have been reviewed in former studies [29], including materials, fabrication processes, and the applications. It can be summarized that the materials for flexible neural probes with micro channels mainly consist of: polyimide, PDMS, parylene, and other biocompatible polymers, which have less of a mismatch in elastic module with tissues than conventional Si or glass-based ones. This is important for long-term implantation to avoid glial scar or neuron death around the device [52]. The fabrication processes employed to form flexible microchannels are developed from the well-developed technologies for MEMS devices, such as the sacrificial layer [46,50] and the bonding technique [26,28,45]. In addition to technical feasibility, three other considerations should be taken into account for the design of microchannels, including the maximum flow rate, the pressure, and the parameters of the microchannel. However, these factors can affect each other and also restrict each other. For instance, in practical application the flow rate, which indicates the dosage of the pharmacy, should be satisfied first:

(1) $$Q=n\frac{\Delta P}{R_{f}}$$

where *Q* is the flow rate, *n* is the number of the microchannels, Δ*P* is the pressure difference between the inlet and the outlet, *R_f_* is the flow resistance of the microchannel. Since flexible devices made of polymers have lower ultimate strength than silicon or metal, pressure applied at the inlet should be under a certain threshold to avoid rupture or irreversible deformation in the microchannel. The maximum pressure should also be taken into account at the design stage according to the yield strength of the material and the structure, as well as the allowable strength associated with the fabrication process such as bonding strength between layers. Only after the maximum flow rate and the allowable pressure are checked, the flow resistance can be determined. For uniform microchannels the flow resistance can be estimated by the following formula:

(2) $$R_{f}=\frac{2\eta LC^{2}}{A^{3}}=\frac{128\eta L}{\pi D^{4}}$$

where *η* is the dynamic viscosity of the fluid, *L* is the length of the microchannel, *C* and *A* are the section perimeter and area of the microchannel respectively, while *D* is the diameter of the microchannel for circular microchannels. Equation (2) can be utilized for the dimensional design of the microchannel. For flexible microchannels, the dimensional design can also be affected by some other factors, such as the inflation caused by the applied pressure, bending of the microchannel caused by the insertion procedure and locomotion of the device or the tissue, which would cause change in the flow resistance.

### 2.3. Electrode–Tissue Materials for Neural Interface

Functional interface possesses the ability to combine physical effects, such as electrical [53], magnetic [54], mechanical [55] or optical [56] stimulations, with modified substrates for further exploration and manipulation of stimuli sensitive cell. The majority of the existing studies have already incorporated conductive biomaterials involving electrical stimulating character as electrode–tissue interface in neural engineering investigations [57,58,59]. Park et al. and Huang et al. reported that the differentiation and maturity of neural stem cells can be promoted by electrical stimulation on graphene plate and carbon nanotube rope, respectively [60,61]. Zhao reported the effects of skeletal myogenesis on comb pattern substrate by adjusting frequency of electrical stimulating [62]. Lee and his colleagues demonstrated that polypyrrole coated nanofibres were able to induce directional growth of neurons [63]. These studies suggest that conducting substrates with variable structure have great potential in modulating excitable cells for neural engineering.

Conducting polymers have also generated much attention, because they possess various distinctive characteristics, including high charge storage capacity, low impedance, excellent plasticity, and volume electrostrictive effect [64,65,66]. Meanwhile, conducting polymers with good biocompatibility are widely applied in biomedical areas, such as biomedical imaging [67], biosensor [68,69], artificial muscle [70], drug release controller [71], cancer biomarker [72] and neural interface [73,74]. One of the most important roles conducting polymers play is in the electrode–tissue interface material for neural engineering, as it can be easily fabricated into multiple structures [75], modified by different doping [76,77] and regulated to undertake electrical stimulation [78,79].

The fundamental properties of conducting polymers including surface morphology, electrical stimulating performance, stability and biocompatibility heavily depend on the characteristics of the negatively charged dopants, which are also termed counterions. For instance, mechanically strong macromolecules have the ability to enhance the stability of conducting polymer composites [80]. Similarly, nano-materials with excellent conductivity, such as carbon nanotubes, are capable of improving the electrical performance of composite film [81]. The conductive polymer electrode–tissue interface, mainly referred to as PEDOT, is combined with water soluble molecules, bio-molecules, carbon nanotube (CNT) and graphene oxide (GO). Thus, as one of the most used electrode–tissue materials, PEDOT has been emphatically discussed.

**Figure 6:**
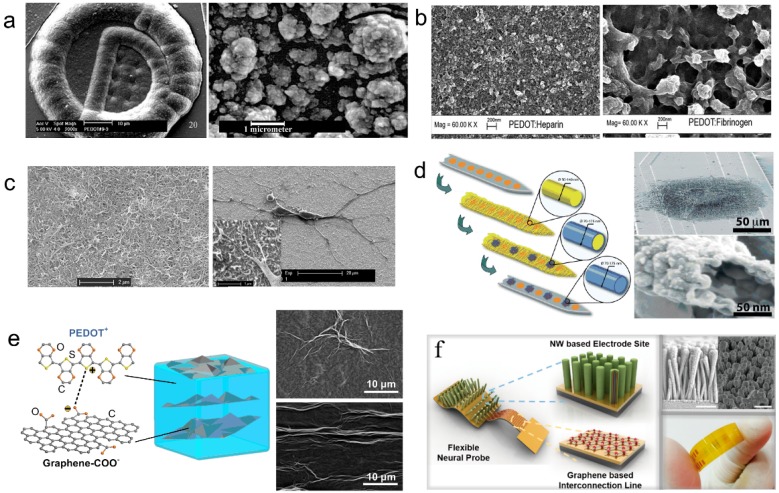
Research progress of the electrode-tissue interface modification technology of flexible MEMS microelectrodes for neural interface. (**a**) PEDOT/PSS composite film, reprinted from [3] with permission of Elsevier, Copyright 2002; (**b**) PEDOT/biomolecules composite film, reprinted from [83] with permission of Springer, Copyright 2008; (**c**) PEDOT/carbon nanotube (CNT) composite film, reprinted from [80] with permission of Elsevier, Copyright 2011; (**d**) PEDOT composite nanotubes, reprinted from [73] with permission of John Wiley and Sons, Copyright 2009; (**e**) PEDOT/graphene oxide (GO) composite film, reprinted from [84] with permission of Elsevier, Copyright 2013; (**f**) PEDOT-Au-ZnO nanowires, reprinted from [87] with American Chemistry Society, Copyright 2017.

Cui et al. of University of Michigan doped polystyrenesulfonate (PSS) as negatively charged counter ion into PEDOT to form the PEDOT/PSS composite as electrode–tissue interface [82]. As exhibited in Figure 6a, the rough and porous structure of the PEDOT/PSS composite greatly increased the effective area of electrode–tissue interface, and improved the electrochemical performance. Therefore, the PEDOT/PSS composite has become one of the most widely used conductive polymer materials for neural interface. Even so, it is not perfect. There are still some problems, such as the long term stability and safety under chronic implantation. For example, Cui et al. of University of Pittsburgh demonstrated some cracking and delamination that were found in PEDOT/PSS film on Pt electrode sites under a biphasic, cathodic first-current pulse stimulation [3]. In their study, thirty electrodes from three electrode arrays were stimulated for stability evaluation. At the end of the two-week stimulation, visual examination was carried out on the electrode surfaces using an optical microscope. There were some minor cracks on the PEDOT layer observed on nine electrodes (30%), and delamination of PEDOT from metal substrate were found on 7 (23%) electrodes. The thickness of the coatings, which is approximately proportional to deposition time, has direct influence on the mechanical failure observed.

Asplund et al. of the Royal Institute of Technology doped hyaluronic acid (HA), heparin and fibrinogen as negatively charged counter ions separately into PEDOT to form PEDOT/bio-molecule composites as electrode–tissue interface [83]. As exhibited in Figure 6b, the three kinds of composite electrode–tissue interface doped with bio-molecules differs from each other in their characteristics, including: surface morphology, effective area determined by surface roughness and thus induced electrochemical performance. The addition of bio-molecule might improve the biocompatibility of the conducting polymer electrode–tissue interface. Furthermore, bio-active drug and nutrition factors could also be added to the PEDOT/bio-molecule composites to produce drug-loaded functionalized electrode–tissue interface.

Luo et al. of the University of Pittsburgh doped acidified carbon nanotube (CNT) as negatively charged counter ion separately into PEDOT to form PEDOT/CNT composite as electrode–tissue interface [80]. As exhibited in Figure 6c, the PEDOT/CNT composite exhibited a rougher surface than the other PEDOT electrode–tissue interface, which facilitated the improvement of electrochemical performance though increasing effective area. The neuron grew well and tightly attached on the PEDOT/CNT composite film, which indicated that the PEDOT/CNT neural interface possessed good biocompatibility. The loose and porous structure of the PEDOT/CNT composite was attributed to the addition of the nano-scale carbon nanotube, which sharply increased the effective area. Moreover, due to the outstanding electrical and mechanical performance, the PEDOT/CNT composite possessed excellent electrochemical properties and stability.

Abidian et al. of the University of Michigan doped perchlorate (ClO_4_^−^) as negatively charged counter ion separately into PEDOT to form hollow nanotube structure as electrode–tissue interface [73]. As exhibited in Figure 6d, the PEDOT nanotube intersected and stacked together to form loose and porous extensional organization on the electrode surface, which improved the effective surface area of electrode–tissue interface. The hollow structure of the PEDOT nanotube further increased the electrode–tissue interface area, which resulted in the improvement of electrochemical performance. 

Tian et al. of Shanghai Jiao Tong University doped graphene oxide (GO) as negatively charged counter ion separately into PEDOT to form the PEDOT/GO nanocomposite film as electrode–tissue interface [84,85,86]. As shown in Figure 6e, in PEDOT/GO composite film, GO disorderly distributed as the structural material to form three dimensional crossover networks, while PEDOT served as stable charge transfer medium was interspersing among the interspaces of graphene nets. Like rebar in concrete, GO doping enhanced the mechanical property of conducting polymer film. Meanwhile, the conducting polymer encapsulation prevents GO from dispersing to the tissue during recording or stimulation process, which greatly abates the possibility of cytotoxicity induced by carbon nano material diffusion while contacting with tissue directly. Like carbon nanotube, the nano-scale graphene oxide also possessed excellent multi-functional properties, which facilitated the improvement of the performance of the conducting polymer electrode–tissue interface.

Ryu et al. of the Daegu Gyeongbuk Institute of Science and Technology demonstrated a neural probe structure based on graphene, ZnO nanowires, and conducting polymer that provides flexibility and low impedance performance [87]. As shown in Figure 6f, the hybrid Au and graphene structure was utilized to achieve both flexibility and good conductivity. The ZnO nanowires was adopted to increase the effective surface area which can drastically decrease the impedance value and enhance the signal-to noise ratio (SNR). The PEDOT coating on the neural probe can also improve the electrical characteristics of the electrode while providing better biocompatibility. In vivo neural signal recordings showed that the neural probe can detect clearer signals.

## 3. Future Development Prospect

Flexible microelectrodes create challenges for the fabrication of ultra-small metal patterns for better resolution. Thus, the development of microelectrodes with tiny dimension (sub-micrometer) and high-density electrode sites will be the goal for researchers. However the high-density electrode sites make the electrical connection difficult, which in turn will necessitate integration of the electronic circuit with the implantable electrode. However, integrating a commercial electronic circuit into a soft substrate is a challenge, since the substrate is flexible and sometimes stretchable. Delamination of the metal layer of chronically implanted soft electrodes is another critical problem [88]. Therefore, integrating electronic circuits into the flexible microelectrodes and improving elasticity and adhesiveness of the metal layer will be another goal for flexible microelectrodes fabrication. Furthermore, new technologies and materials (such as shape memory materials and biodegradable materials) that can assist insertion of the flexible microelectrodes into brain tissue are quite desirable [38,42]. In addition, transparent flexible MEMS microelectrodes facilitate fluorescence observation of neural tissue [37] and stretchable flexible MEMS microelectrodes for conformal covering on brain tissue will become a new direction in this research area. Also, in terms of electrode modifications for electrode–tissue interface, an ideal tissue engineered interface proposed by Aregueta-Robles et al. that incorporated combined coating approaches of conductive polymers, hydrogels and attachment factors with neural cells, should be able to give considerations to each of the requirements of electrode–tissue interface [89].

In recent years, the applications of optogenetics in neuroscience also attracted much attention from neuroscientists. Although electrical stimulation exhibited remarkable efficacy in controlling and exploring the function of discrete brain regions and providing therapeutic solutions, it was unable to target genetically specified neuron types. The disadvantage of electrical stimulation could be overcome with genetically encoded opsins [90]. As a consequence, flexible MEMS microelectrodes integrated with optical stimulation capability for neural interface will be point of special interest in future research.

Other than that, the integration of flexible MEMS microelectrodes for neural interface and other flexible sensors, such as flow rate sensors, will also be promising in future research. Single element or single function devices will not satisfy the requirements anymore. Devices with multiple sensors, which employ electrochemical impedance methods that are compatible with neural probes [91], will be used as they are more convenient for obtaining information (a single device, at the same time). In brief, flexible MEMS microelectrodes integrated with sensors are in great demand.

In addition, the newly rising nanotechnology and biomedical engineering will open a new gate for the development of flexible MEMS microelectrodes for neural interface. The interdisciplinary research of micro fabrication technology with nanotechnology and biomedical engineering will direct future research [92,93,94,95]. The combination of these cutting-edge subjects will undoubtedly collide to make great scientific strides and greatly influence people’s daily lives and understandings of the world.
